# Protein Crystallization in a Microfluidic Contactor with Nafion^®^117 Membranes

**DOI:** 10.3390/membranes11080549

**Published:** 2021-07-21

**Authors:** M. Polino, H. S. Rho, M. P. Pina, R. Mallada, A. L. Carvalho, M. J. Romão, Isabel Coelhoso, J. G. E. Gardeniers, J. G. Crespo, Carla A. M. Portugal

**Affiliations:** 1 LAQV-REQUIMTE, Department of Chemistry, NOVA School of Science and Technology, Universidade Nova de Lisboa, 2829-516 Caparica, Portugal; mariella.polino@gmail.com (M.P.); imrc@fct.unl.pt (I.C.); jgc@fct.unl.pt (J.G.C.); 2 Department of Instructive Biomaterials Engineering, MERLN Institute for Technology-Inspired Regenerative Medicine, Maastricht University, 6229 ER Maastricht, The Netherlands; h.rho@maastrichtuniversity.nl; 3 Instituto de Nanociencia y Materiales de Aragón (INMA), Universidad de Zaragoza-CSIC, 50009 Zaragoza, Spain; mapina@unizar.es (M.P.P.); rmallada@unizar.es (R.M.); 4 Department of Chemical & Environmental Engineering, University of Zaragoza, 50018 Zaragoza, Spain; 5 Networking Research Centre on Bioengineering, Biomaterials and Nanomedicine, CIBER-BBN, 28029 Madrid, Spain; 6 UCIBIO–Applied Molecular Biosciences Unit, Department of Chemistry, School of Science and Technology, NOVA University Lisbon, 2819-516 Caparica, Portugal; almc@fct.unl.pt (A.L.C.); mjr@fct.unl.pt (M.J.R.); 7 Associate Laboratory i4HB, Institute for Health and Bioeconomy, School of Science and Technology, NOVA University Lisbon, 2819-516 Caparica, Portugal; 8 Mesoscale Chemical Systems, TNW Faculty, University of Twente, P.O. Box 217, 7500 AE Enschede, The Netherlands

**Keywords:** Nafion^®^ membrane, membrane contactors, protein crystallization, solute diffusion, protein structure

## Abstract

Protein crystallization still remains mostly an empirical science, as the production of crystals with the required quality for X-ray analysis is dependent on the intensive screening of the best protein crystallization and crystal’s derivatization conditions. Herein, this demanding step was addressed by the development of a high-throughput and low-budget microfluidic platform consisting of an ion exchange membrane (117 Nafion^®^ membrane) sandwiched between a channel layer (stripping phase compartment) and a wells layer (feed phase compartment) forming 75 independent micro-contactors. This microfluidic device allows for a simultaneous and independent screening of multiple protein crystallization and crystal derivatization conditions, using Hen Egg White Lysozyme (HEWL) as the model protein and Hg^2+^ as the derivatizing agent. This microdevice offers well-regulated crystallization and subsequent crystal derivatization processes based on the controlled transport of water and ions provided by the 117 Nafion^®^ membrane. Diffusion coefficients of water and the derivatizing agent (Hg^2+^) were evaluated, showing the positive influence of the protein drop volume on the number of crystals and crystal size. This microfluidic system allowed for crystals with good structural stability and high X-ray diffraction quality and, thus, it is regarded as an efficient tool that may contribute to the enhancement of the proteins’ crystals structural resolution.

## 1. Introduction

The attainment of high-quality diffracting crystals is still the main limitation in protein crystallography, the most employed method for the determination of the three-dimensional structure of proteins. The diffraction quality is dependent on the internal order of the crystal lattice. To obtain well-ordered crystals, several crystallization parameters such as pH, temperature, solvent removal rate, and additives need to be assayed. Therefore, when the structure of a new protein needs to be unraveled, an enormous number of pre-formulated conditions are commonly screened, before finding those that may lead to crystals with the quality needed for an accurate crystallography analysis [1].

Microfluidic technology has been revolutionary for protein crystallization. The creativity of scientists has led to the development of several intricate chip designs (valve-based [2], droplet-based [3], slip chips [4], or centrifugal designs [5]) that allowed for the fast screening of hundreds of process conditions, using only very low amounts of protein [6], in devices that can be mounted in front of an X-ray beam, allowing for diffraction screening without crystal handling [7]. 

On the other hand, advances in membrane technology have been driving the development of membranes with precisely tailored characteristics, either by modulation of the porosity of hydrophobic microporous membranes, e.g., polypropylene, or by controlling the difference in water activity between the protein and the stripping solutions [8]. These attributes contributed to an improved control over the solvent removal rate, which is essential for regulation of the crystallization process. Membrane properties have shown to be determinants for the control of crystal growth rates [9], shape [10], polymorphism [11] and, consequently, the crystals’ diffraction quality [12]. 

In some cases, the 3D structure of novel proteins and specific protein structural folds cannot be solved using the molecular replacement method, the incorporation of heavy atoms into the crystal (derivatization) being necessary so that isomorphous replacement (MIR—Multiple Isomorphous Replacement, SIR—Single Isomorphous Replacement, MIRAS—Multiple Isomorphous Replacement with Anomalous Scattering, or SIRAS—Single Isomorphous Replacement with Anomalous Scattering) methods can be applied [13,14,15,16,17]. Crystal derivatization is also a challenging procedure as the identification of the right heavy atom and concentration for a specific protein may correspond to laborious work, requiring persistence. Furthermore, crystal derivatization commonly involves the immersion of crystals into the derivatization media, leading to easier crystal cracking and damaging due to the use of heavy atom compounds that are too reactive or due to abrupt changes in the local growth environment. Attempts were made to predict the interaction between the protein and the heavy atoms [18,19], but in most cases, a previous screening of different heavy atoms and concentrations becomes essential. 

We have recently described how ion-exchange membranes can be used to facilitate the derivatization of protein crystals with heavy atoms [20]. Ion-exchange membranes are typically made of a hydrophobic backbone with attached charged groups [21]. Such membranes are able to mediate the selective diffusion of ions (cations or anions depending on the type of fixed charge groups attached to the polymer backbone) to the protein crystal solution, leading to a smooth and controlled increase in the concentration of the target ion, thus reducing the risks of cracking due to abrupt changes in the crystal environment and crystal handling [20]. Besides the selective ion transport, ion-exchange membranes promote water transport when a difference in water activity occurs between the two sides of the membrane. In this case, water spontaneously moves from the least to the most concentrated compartment [22]. Hence, controlled diffusion of water by osmosis could be exploited to generate supersaturation and promote nucleation.

Herein, a commercial ion-exchange membrane (117 Nafion^®^) was integrated into a PDMS (polydimethylsiloxane) microdevice to form 75 micro-contactors. The microdevice consisted of two independent chambers (a wells layer dedicated to the crystallization solution and a channels layer filled with stripping/derivatization solution) separated by an ion-exchange flat membrane. In particular, each well in the crystallization chamber can accommodate nano- or microliter volumes of protein solution, defining the area for water transport and ion-exchange, whereas the transport rate of the different species in solution is controlled by the ion-exchange 117 Nafion^®^ membrane. In addition, due to miniaturization, the volume of protein crystallization/derivatization solutions required in the well/channel chambers is notably reduced while providing high throughput for the preliminary screening of different crystallization/derivatization operational conditions. Thus, the final aim of this work was to demonstrate the feasibility of the ion-exchange membrane-driven crystallization process in a dedicated Nafion^®^-based microfluidic device. In this regard, this work highlights the contribution of this microfluidic device for improvement of protein crystallography processes, by promoting the needed conditions for fast formation of protein crystals with high diffraction quality, which is an essential requirement for solving the three-dimensional structure of proteins. The crystallization performance, i.e., growth rate, size, and diffraction quality of crystals, was evaluated using Hen Egg White Lysozyme (HEWL) as a protein model.

## 2. Materials and Methods

### 2.1. Crystallization Solutions

A sodium acetate buffer at 0.1 M and pH 4.6 was prepared using CH_3_COONa (Scharlab S.L., Barcelona, Spain). Lyophilized Hen Egg White Lysozyme (HEWL) from Sigma Aldrich was dissolved in 0.1 M of CH_3_COONa at a concentration of 50 mg/mL (protein solution). NaCl (Applichem Panreac, Barcelona, Spain) was dissolved in 0.1 M of CH_3_COONa (pH 4.6) to a concentration of 3.5% *w*/*v* (precipitant solution). The protein and the precipitant solutions were mixed in equal volumes in order to obtain a starting crystallization solution composed of 25 mg/mL of HEWL, 1.75% (*w*/*v*) NaCl, and 0.1 M of CH_3_COONa at pH 4.6. A solution of 3.5% (*w*/*v*) NaCl in 0.1 M of CH_3_COONa at pH 4.6 was used as the stripping agent in the channels.

One of the most used heavy atoms in protein crystals derivatization is mercury. Indeed, thanks to its high atomic number (hence, high number of electrons) and the ability to establish covalent bonds with cysteine residues, it facilitates the derivatization process with proteins containing these types of residues such as Lysozyme. Mercury can be available for derivatization in a wide range of compounds, such as Hg(CH_3_COO)_2_, which has the advantage of relatively good solubility in 0.1 M of CH_3_COONa buffer (conventionally used for the crystallization of Lysozyme) compared to other derivatizing agents (such as HgCl_2_) [13].

Hg(CH_3_COO)_2_ in solution can be found in the ionic form of CH_3_COO^−^ and Hg^2+^. Hence, in order to model the diffusion of Hg^2+^ through the membrane, measurements of the mass transfer coefficient of Hg^2+^ to be eventually used for derivatization were performed by using Hg(CH_3_COO)_2_ dissolved in a solution containing 0.1 M of CH_3_COONa at pH 4.6 and 0.59 M of NaCl.

### 2.2. Design and Fabrication of the Microdevice

**Figure 1:**
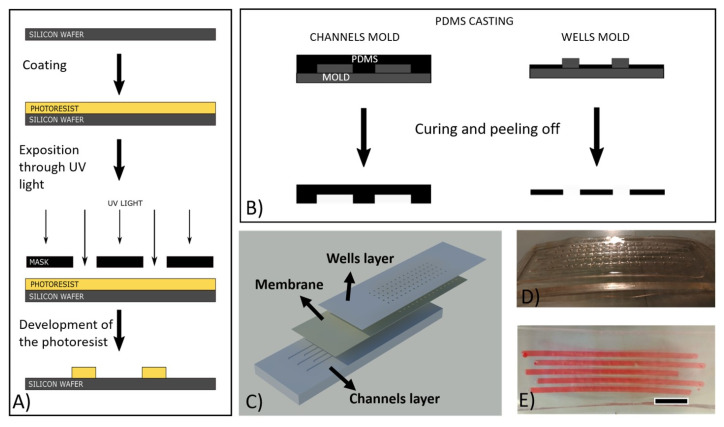
(**A**) Mold fabrication by photolithography process: SU8 photoresist deposition on Si wafer; exposition of the designed mask to UV light; development of the SU8 photoresist to attain the final mold. (**B**) The SU8 molds were used for the fabrication of both PDMS compartments by casting. (**C**) AutoCAD image showing the different components of the microdevice, i.e., channel layer (on the bottom), membrane (in between layers), and the wells layer (at the top). (**D**) Photo of the fabricated microdevice. (**E**) Photo showing a top view of the fabricated microdevice (the channel layer was filled with a molecular dye solution to highlight the presence of the channels, their geometry, and dimension. The scale bar in figure (**E**) corresponds to 1 cm.

The microdevice was fabricated by soft lithography [23,24,25]. Two photomasks, one for a microwell layer and another one for a channel layer, were designed using CleWin software (WieWeb software, Hengelo, The Netherlands). Master molds were fabricated by standard photolithography (Figure 1A) [26]. A negative photoresist resin (SU-8 2150, MicroChemicals GmbH, Ulm, Germany) was spun onto 4” Si wafers, baked, and exposed to UV light in order to transfer the pattern from the mask to the photoresist layers on the wafers. The subsequent use of an SU-8 developer allowed for the removal of the soluble (nonexposed) parts of the resin. The final thickness of the photoresist structures was measured with a Veeco Dektak 8 surface profiler and it was found to be 300 ± 15 µm for both molds.

Polydimethylsiloxane (PDMS) mixture (Sylgard 184, Dow Corning, prepolymer: curing agent = 10:1) was casted onto the master molds and baked at 80 °C for 1 h (Figure 1B). For the channel layer, an amount of PDMS was casted to cover the mold completely (Figure 1B). Instead, in the case of the wells layer, the volume of PDMS casted was calculated in order to give a thickness lower than the height of the pillars, determining the formation of holes, instead of cavities (Figure 1B). In order to flow the solutions inside the channels, an inlet and an outlet were created by punching. Each device had 5 lines of 15 wells for a total of 75 wells. The wells had a circular shape 1 mm in diameter (this diameter was chosen to allow the harvesting of crystals with conventional crystallography loops) and 250 µm in depth. The channel compartment comprised 5 channels (with a width of 2 mm and a depth of 300 µm), matching with the 5 lines of wells; therefore, 5 different solutions can be used simultaneously as stripping solutions (1 per channel) for crystallization. The driving force in each channel would be dependent on the solution inside the wells. The same channels may be later used to circulate the solutions selected for crystal derivatization. An AutoCAD image (Autodesk, San Rafael, CA, USA) rendering the three layers of the device is shown in Figure 1C; photos of the fabricated device are shown in Figure 1D (cross-section) and Figure 1E (top-view). 

A 117 Nafion^®^ membrane (Sigma-Aldrich, 1100EW, St. Louis, MI, USA) was sandwiched between the channel and the well layers. Nafion^®^ is a material with a high degree of swelling; therefore, the bonding with PDMS was quite challenging. Several procedures are described in the literature [27,28,29,30], and the protocol developed by Pham et al., for the commercial 117 Nafion^®^ membrane, was elected and used in this work after optimization [31]. Briefly, the Nafion^®^ membrane was cleaned in 3% H_2_O_2_ at 80 °C for 1 h, H_2_O at 80 °C for 1 h, 1 M of H_2_SO_4_ at 80 °C for 1 h, and H_2_O at 80 °C for 1 h. The membrane was dried at 80 °C for 24 h and then treated for 15 min at 150 °C in order to reduce the swelling behavior. It has been reported that the thermal treatment of Nafion^®^ membranes may induce conformational changes and spatial reorientation of the hydrophobic and hydrophilic nanodomains, leading to a lower water uptake and conductivity [32]. The washed and thermally treated 117 Nafion^®^ was modified with a corona discharge (BD-20AC Laboratory Corona Treater, Chicago, IL, USA) for 10 min in order to generate hydroperoxide groups. Previous trials were made with plasma oxygen equipment; however, the strong vacuum led to a severe shrinkage of the membrane that turned to be too wavy to create good contact with the modified PDMS surface.

The PDMS layers were treated with oxygen plasma for 60 s, in order to form hydroxide groups, and then immersed in 4% triethoxyvinylsilane (VTES) (purchased from Sigma-Aldrich, 97%) in ethanol (Honeywell, purity ≥98.8%) with 10% of water for 2 min and baked at 100 °C for 15 min to allow grafting to occur. 

Afterward, the treated 117 Nafion^®^ membrane was contacted with the grafted PDMS and baked at 100 °C for 2 h to promote the formation of radical groups on the membrane, which would attach to the vinyl group in PDMS-VTES and form the bonding. 

After the bonding, the microdevice was soaked in 2 M of NaCl solution in order to exchange acidic groups with Na+ and avoid pH changes in the crystallization solution that may induce crystals’ cracking and/or dissolution. The NaCl solution was replaced until the pH of the solution was kept neutral to ensure that all protons were exchanged for Na+.

### 2.3. Crystallization Experiments

**Figure 2:**
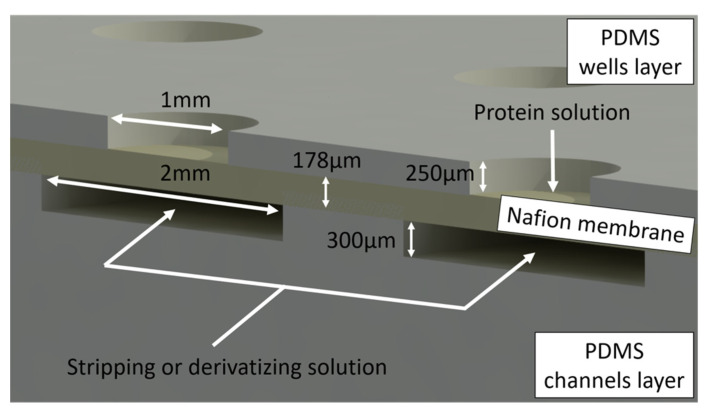
Cross-section scheme of the microdevice.

Crystallization experiments were performed in order to confirm the ability of the microdevice to produce crystals. First, the channels of the device were filled with the stripping solution (3.5% *w*/*v* NaCl dissolved in 0.1 M of CH_3_COONa) using a syringe pump, and, later, the wells were filled with the protein solution using a micropipette (Figure 2). Mainly, three different volumes of protein solution were used: 500 nL, 1 μL, and 2 µL, for the same membrane area. Each condition was repeated at least 9 times for assessing the experimental reproducibility. Finally, the chip was placed in a sealed box to prevent evaporation, in a room with controlled temperature (20 °C).

### 2.4. Modeling of Water and Hg^2+^ Transport through 117 Nafion^®^ Membrane

From structural investigations of the Nafion^®^ ionomer, it is well-known that the hydrophilic sulfonic groups organized in clusters can incorporate water and allow for ions/protons and water transport. Accordingly, these properties were exploited, aiming to remove water from the protein solution in order to achieve local supersaturation and facilitate nucleation. The driving force for water transport in the microdevice was established by filling the channels with a stripping solution with a lower water activity compared to the protein solution placed in the wells (more details are reported in the ‘Crystallization experiments’ section). Note that, although water may permeate PDMS (microdevice material), the water diffusion through the PDMS walls of the microdevice is considered negligible, as the contacting area of the protein solution with the PDMS material adjacent to the wells is much smaller than that with the Nafion^®^ membrane. Furthermore, the hydrophobic polytetrafluoroethylene (PTFE) backbone of Nafion^®^ contains negatively fixed charge groups, which enable the selective transport of cations.

In order to evaluate the transport of water through the 117 Nafion^®^ membrane and estimate the variation in NaCl concentration, a diffusion cell was set-up to mimic the conditions of the crystallization environment in the micro-device.

**Figure 3:**
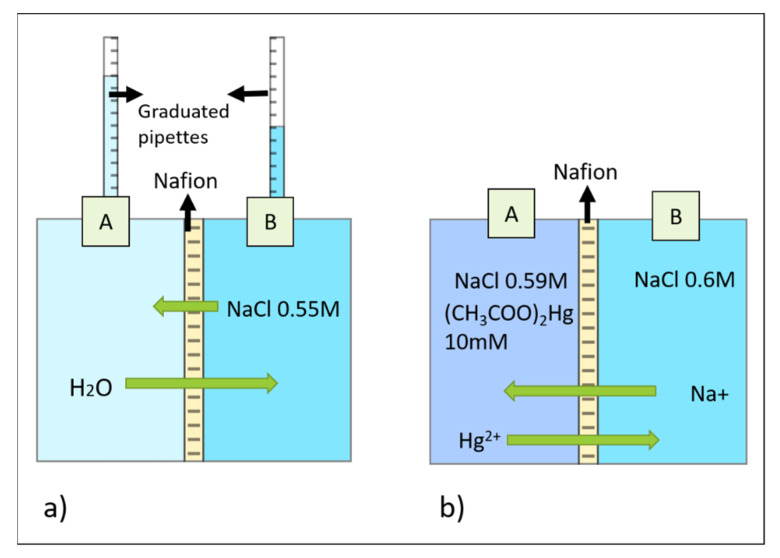
(**a**) Diffusion cell used to measure water and Na^+^ mass transfer coefficients in Nafion^®^; (**b**) diffusion cell used to measure Hg^2+^ mass transfer coefficient in Nafion^®^.

The diffusion cell, sandwiching the 117 Nafion^®^ membrane (previously hydrated) between two compartments, compartment A and B, is shown in Figure 3a. Compartment A was filled with distilled water and compartment B was filled with a 0.55 M of NaCl solution in order to create the driving force for mass transport (ions and/or water transport) to occur. Two graduated pipettes were connected to the outlet of each compartment to record changes in the volume as a function of time. In this situation, no ion-exchange process occurred, due to the absence of a cation to be exchanged in compartment A with the Na^+^ available in compartment B. However, a leak of NaCl due to the high osmotic pressure might still be possible [33]. In order to assess the extent of the leak and the variation in driving force within the monitoring time, the conductivity of the solutions was evaluated and the results are reported in Figure S1 in the Supporting Information (S.I.). 

The selective transport of cations promoted by the membrane might instead be exploited for a controlled diffusion of ions to/from the protein crystals solution, as well as for a gentle crystal derivatization process. Derivatization of protein crystals commonly takes place after crystal formation in order to maintain the isomorphism [13]. Hence, when derivatization is performed, the composition of the protein crystal solution is already equilibrated with the stripping solution as they have the same osmotic pressure. Therefore, in order to investigate the transport of cations for derivatization in the microdevice, a second diffusion cell (shown in Figure 3b) was set-up, in which conditions for crystal derivatization were simulated. The diffusion cell was used to calculate the mass transfer coefficient of Hg^2+^ across the membrane (previously equilibrated in a NaCl solution). Hg^2+^ is commonly used for the derivatization of protein crystals due to its ability to form covalent bonds with cysteine residues [13] and, thus, it was selected to prove the concept of this work. Two solutions with the same osmotic pressure were used. Compartment A was filled with a solution containing 0.59 M of NaCl and 0.01 M of Hg(CH_3_COO)_2_, whereas Compartment B was filled with a 0.6 M of NaCl solution. Na^+^ and Hg^2+^ were then allowed to exchange until they reached equilibrium. Samples were taken over time, and the concentration of Hg^2+^ in Compartment B was measured by an ICP−AES (inductively coupled plasma−atomic emission spectrometer, Horiba Jobin-Yvon, France). The concentration of Hg^2+^ corresponded to the average value of three replicates, with an associated error <5%. 

### 2.5. X-ray Diffraction Analysis and Structure Determination

Previous to X-ray diffraction analysis, HEWL crystals were equilibrated for a few seconds, first in a harvesting buffer (0.1 M of CH_3_COONa, pH 4.6, and 1 M of NaCl) and then in a cryo-protectant solution (harvesting buffer with 30% (*v*/*v*) glycerol from Sigma-Aldrich). 

X-ray diffraction analysis was performed using an in-house X-ray diffractometer (IμS 3.0 microfocus D8 Venture from Bruker, with CuK_α_ radiation), coupled to a CMOS Photon 100 detector, at 110 K. Indexing and integration were performed using a PROTEUM3 software pipeline (Bruker AXS 2015). Scaled and merged intensities were converted to amplitudes using the COMBAT program from the CCP4i suite [34]. The structure was solved via molecular replacement (MR). MR is a method widely used in protein crystallography where the structure of a previously studied protein containing similarities with the structure of the protein under investigation is used as a model for recovering the phase information lost during X-ray diffraction (phase problem [13,14,35]) and to resolve the structure [36]. 

HEWL is widely studied in the literature; hence, the structure of a previously resolved HEWL molecule (3A8Z available at the protein data bank PDB [37]) has been used as a search model. Phases were calculated using the Expert MR-PHASER function from CCP4i2 suite. Model building and refinement were performed, iteratively, using COOT [38] and REFMAC5 [39]. A final model was built using BUCCANEER [40] and viewed in CCP4mg [41]. The MOLPROBITY program [42] was used for the validation of the final model. 

## 3. Results and Discussion

### 3.1. Estimation of Water and Hg^2+^ Transport across Nafion^®^ Membrane

The water mass transfer coefficient was used to estimate the variation in salt concentration in the protein solution due to osmosis. The mass transfer coefficient of Hg^2+^ and NaCl were used to estimate the NaCl and Hg^2+^ concentration profiles over time in the protein crystals solution during the crystal derivatization process.

**Figure 4:**
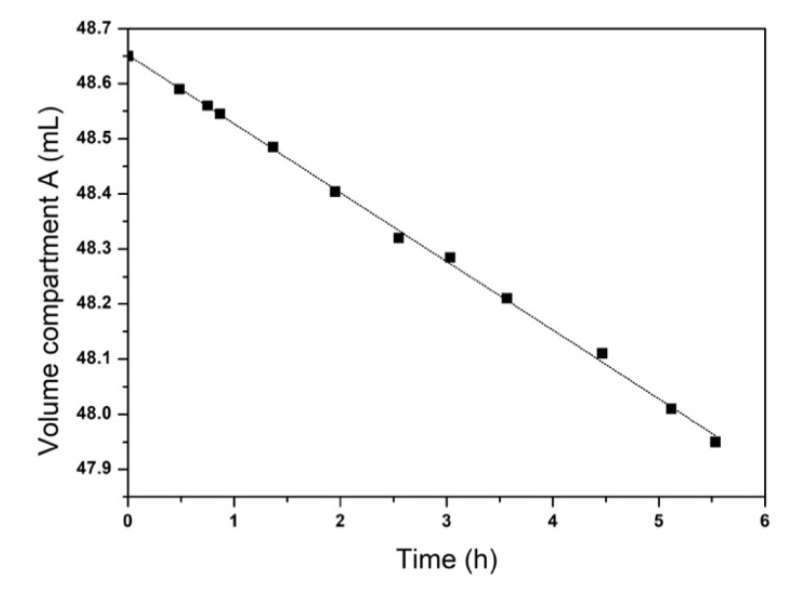
Volume of water over time in compartment A of the diffusion cell in Figure 3a. Volume measurement error <1%.

When a cation-exchange membrane (as Nafion^®^) contacts a pure water solution on one side and a salt solution on the other side, water moves from the water compartment to the salt solution compartment until the osmotic pressure is equilibrated. In order to calculate the mass transfer coefficient of water, a previously hydrated Nafion^®^ membrane was placed in the diffusion cell (2a) and the variation in volume was followed over time in the two compartments (Figure 4). At the beginning of the osmosis process, the volume decreased linearly with time in compartment A, with a slope of 0.125 mL/min, which corresponds to the volumetric flow rate of water across the membrane ($Q_{w}$).

From $Q_{w}$, it is possible to calculate the molar flux of water, $J_{w}$, considering the values of density (d), molecular weight ($M_{w}$) of water, and the membrane area (A) (7.54 cm^2^), as follows:

(1) $$J_{w}=\frac{Q_{w}d}{AM_{w}}$$

From $J_{w}$, the mass transfer coefficient $K_{w}$ was calculated as follows:

(2) $$K_{w}=\frac{J_{w}l}{\left(∆p-∆\pi \right)}$$

where ∆p is the hydrostatic pressure difference, ∆π is the osmotic pressure difference, and l is the membrane thickness (178 µm). The hydrostatic pressure was considered negligible, whereas ∆π was calculated as ∆π=∆CRT, where ∆C is the molar concentration difference of NaCl in the two compartments (the short measuring period ensured a very small variation in driving force, i.e., 4%, with details reported in Figure S2 in the Supporting Information). Hence, an average concentration value within the measurement interval was used for this calculation (0.52 M), where R is the ideal gas constant ($0.08206\frac{L\;atm}{mol\;K}$) and T is the temperature (298.15 K).

**Figure 5:**
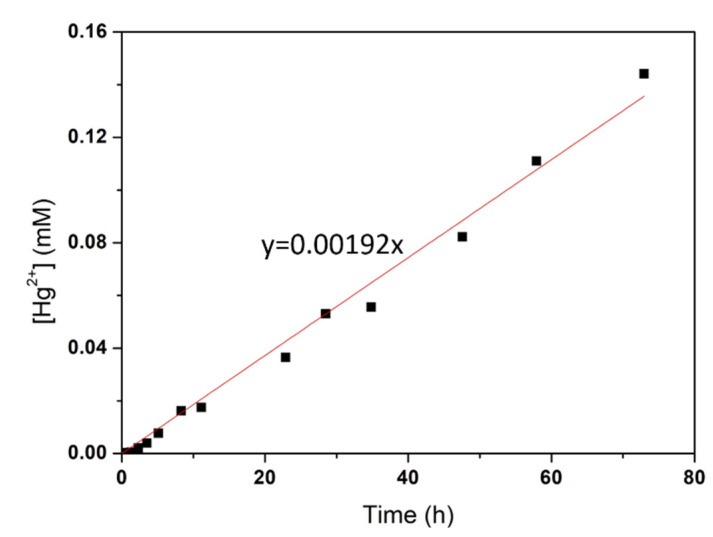
Hg^2+^ concentration over time in compartment A of the diffusion cell. The concentration of Hg_2_^+^ was determined by ICP-AES with an associated error <5%.

The concentrations of Hg^2+^ over time was also measured in the cell shown in Figure 3b for a membrane already equilibrated in a NaCl solution in order to determine the mass transfer coefficient of this ion. In this case, the osmotic pressure on the two sides at the beginning of the experiment was the same. However, the charge difference between Hg^2+^ and Na^+^ leads to the exchange of 2 Na^+^ cations for each Hg^2+^ cation, to maintain the electroneutrality, changing the osmotic equilibrium between the two solutions. In order to reinstate the osmotic equilibrium, some water might have crossed the membrane. However, as the amount of Hg^2+^ used here was very small (10 mM), comparatively to the concentration that was responsible for the total osmotic pressure (0.7 M), the water transport was negligible. For this reason, the volume of the solutions in compartments A and B (on the two sides of the membrane) was considered constant. Taking this into account, the molar flux ($J_{Hg}$) was calculated by dividing the slope of the curve (0.00192 mM/h) in Figure 5 by the area of the membrane (A) and multiplying by the volume (V) (Equation (3)):

(3) $$J_{Hg}=\frac{mol_{Hg}}{tA}$$

The $J_{Hg}$ can be also defined as:

(4) $$\;J_{Hg}=K_{Hg}∆C$$

where $K_{Hg}$ is the mass transfer coefficient and ∆C is the Hg^2+^ concentration difference between the two sides of the membrane (i.e., between compartments A and B) that was considered constant because of its small value (10 mM). Hence, $K_{Hg}$ was calculated as:

(5) $$K_{Hg}=\frac{J_{Hg}}{∆C}$$

The mass transfer coefficients of water and Hg^2+^ through the Nafion^®^ membrane are compared in Table 1. The low mass transfer coefficient of Hg^2+^ indicates a slow diffusion of this cation through the membrane, which is an excellent characteristic considering the need to promote a gentle derivatization process.

The same rational was used to determine the concentration profile of NaCl in compartment A and the transport of NaCl through the membrane over time. These data are shown in Figures S3 and S4, in the S.I., respectively.

### 3.2. Simulation of Transport in the Microdevice

**Figure 6:**
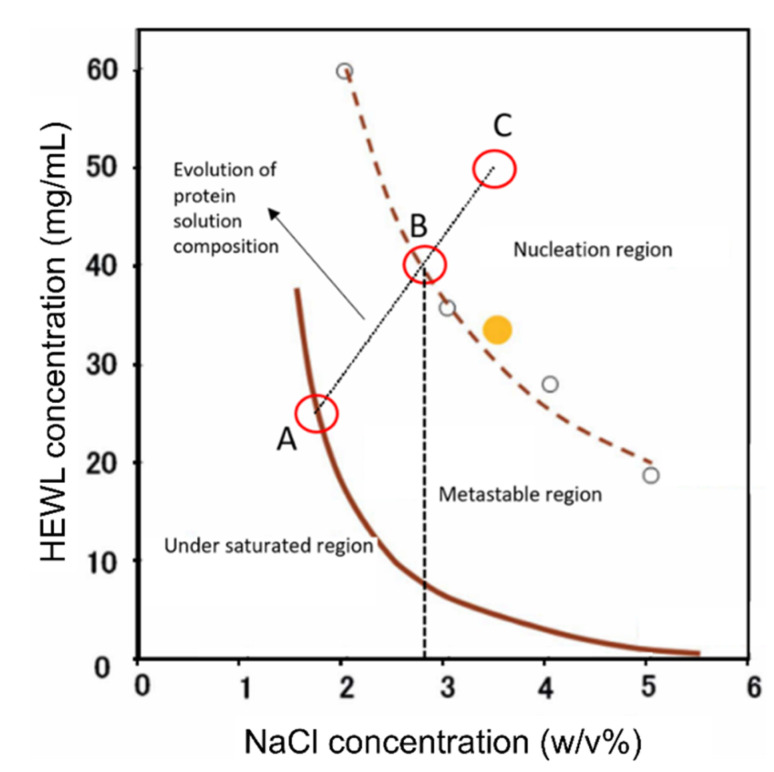
Solubility diagram of lysozyme extracted adapted from [43]. (**A**) corresponds to the composition of the crystallizing solution in the beginning of the experiment (HEWL 25 mg/mL and NaCl 1.75% (*w*/*v*)); (**B**) corresponds to the composition of the crystallizing solution when crossing the boundary for nucleation to occur (HEWL 41 mg/mL, NaCl 2.9% (*w*/*v*)); (**C**) corresponds to the equilibrium point with the stripping solution (HEWL 50 mg/mL, NaCl 3.5% (*w*/*v*)). The brown solid curve corresponds to the saturation boundary line, the brown dashed curve corresponds to the nucleation boundary line, and the dashed black vertical line shows the NaCl concentration in the crystallizing solution in the moment nucleation begins.

**Figure 7:**
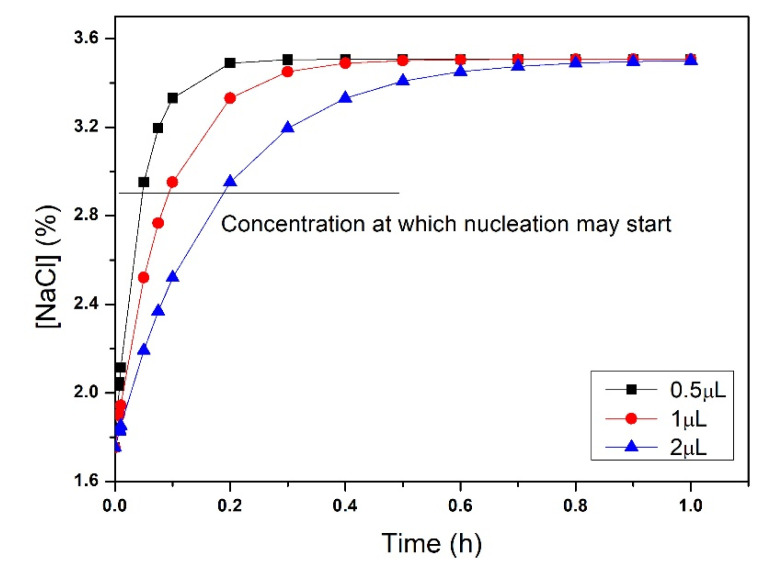
NaCl concentration in the wells of the micro-device over time for different volumes of protein solution.

Crystallization experiments in the microdevice were performed using the widely investigated model protein lysozyme (Hen Egg White Lysozyme—HEWL). Crystallization conditions for HEWL can be found in the phase diagram of the protein [43]. The phase diagram of HEWL combined with simulations of the evolution of the salt concentration in the micro-device was used to predict when the nucleation conditions were reached. The evolution of the initial composition of protein solution (protein concentration: 25 mg/mL and NaCl concentration: −1.75% (*w*/*v*)) to the final concentration equilibrated with the stripping solution (protein concentration: 50 mg/mL and NaCl: −3.5% (*w*/*v*)) was overlaid on the phase diagram in Figure 6. It is possible to notice that when the salt concentration was about 2.9%, the solution was supersaturated at a level where nucleation is likely to occur. By using the calculated mass transfer coefficient of water (Table 1) and the geometric dimensions of the device, it was possible to simulate well the variation in NaCl concentration in the protein over time, when a stripping solution of 3.5% (*w*/*v*) NaCl was used in the channels to promote osmosis. Results of the simulation are reported in Figure 7.

The experimental simulation was run considering three different solution volumes (V_1_ = 0.5 µL, V_2_ = 1 µL, V_3_ = 2 µL) and the same area (A_wells_ = 7.85 cm^2^) for mass transport. The time at which nucleation may start was highlighted, and the nucleation condition was reached in a short fraction of an hour, for the three different volumes, meaning that the nucleation kinetics was very fast.

**Figure 8:**
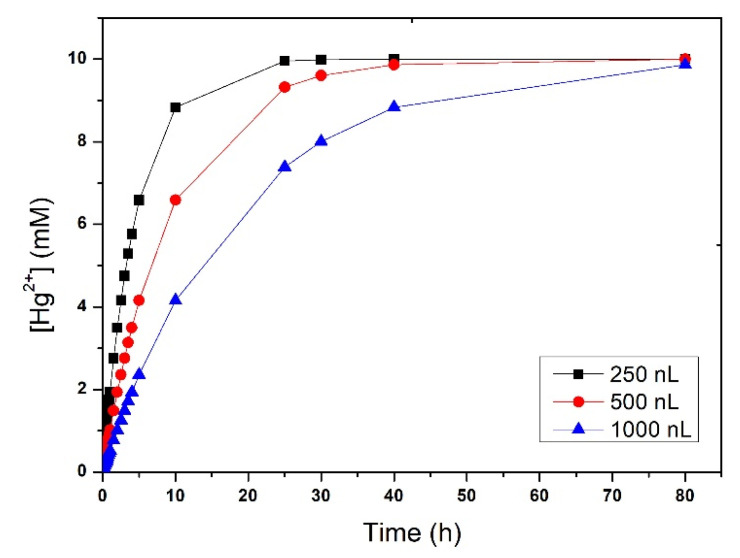
Evolution of Hg^2+^ concentration in the protein solution.

In order to investigate the impact of Hg^2+^ on the crystals, a simulation was run in order to estimate the increase in Hg^2+^ concentration in the wells (Figure 8). The protein solution deposited in the wells at the beginning of the experiments had a NaCl concentration of 1.75% (*w*/*v*). Instead, the solution used as a stripping phase, in the channels, had a NaCl concentration of 3.5% (*w*/*v*).

Taking into consideration that the buffer type, concentration, and pH (CH_3_COONa 0.1 M, pH 4.6) were the same in both protein and stripping solutions, and that the contribution of the protein molecules to the osmotic pressure was negligible, the protein solution is expected to present an osmotic pressure that is half of the stripping solution. The channels volume (~33 μL) was significantly higher than the volume of the solution placed in the wells (0.5–2 μL). Therefore, during the osmosis process, the change in concentration in the channels is expected to be minimal while the solution in the well would tend to balance the concentration with that in the channel. As equilibrium was reached mainly by water transport, the volume at equilibrium in the wells would be half of the initial volume. 

The crystal derivatization with Hg^2+^ would be performed only when crystallization is completed [18]. For this reason, the volumes used for the calculation of the increase in Hg^2+^ concentration in the wells were half of the initial volumes. Considering these conditions, the maximum cation concentration was reached in about 20 h for 250 nL, 40 h for 500 nL, and about 80 h for 1 µL of the solution. Such long diffusion times will allow gentle transport of the derivatized ions, reducing the risk of crystal cracking and damage during the process. Furthermore, the different ion concentration–time dependences between the three volumes might be useful for controlling the stability of the crystals and the crystal derivatization efficiency. Crystal derivatization experiments in the microdevice are in progress; however, they are not within the scope of this paper.

### 3.3. Crystallization of HEWL in the Microfluidic Device

**Figure 9:**
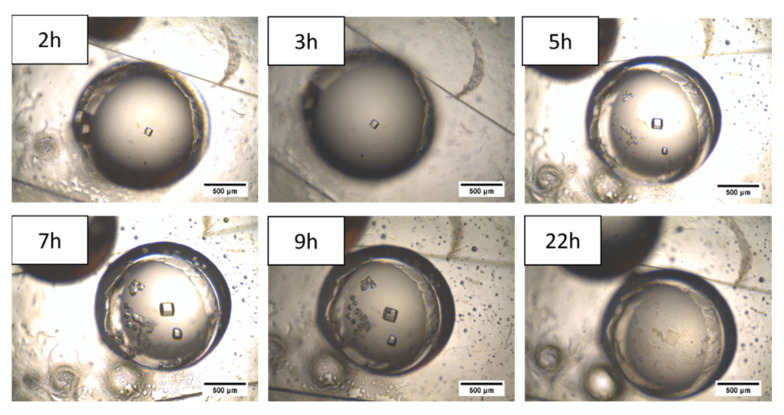
HEWL crystals evolution in the well-type micro-contactor when using the protonated 117 Nafion^®^ membrane.

The first crystallization experiments in the microdevice revealed the formation of HEWL crystals after a short time (2 h), in accordance with the simulations in Figure 7. However, they quickly degraded until complete disappearance (22 h, Figure 9). This empirical observation was attributed to the H^+^/Na^+^ exchange process between the protonated 117 Nafion^®^ membrane and the protein solution. Consequently, the pH gradually decreased to an extreme condition unbearable for the crystals, promoting its degradation. 

**Figure 10:**
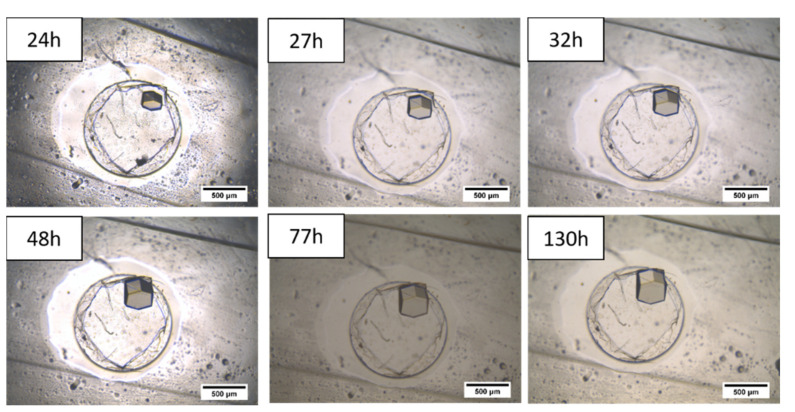
HEWL crystals evolution in the well-type micro-contactor when using the 117 Nafion^®^ membrane in its sodium form.

In order to avoid this inconvenience, and once the chip bonding was unsuccessful when the Na-exchanged 117 Nafion^®^ membrane was used (demonstrated in preliminary results not shown here), the microfluidic chip obtained after the PDMS-Nafion^®^ assembly (described in Section 2.2 “Design and fabrication of the microdevice”) was soaked in a 2 M NaCl solution to obtain the 117 Nafion^®^ membrane in its sodium form. During the H^+^/Na^+^ exchange process, the pH of the solution was monitored over time, and the NaCl solution was periodically replaced until the pH reached a neutral and constant value. Once the microfluidic chip was equilibrated, HEWL crystallization experiments were repeated. Pictures of the crystals obtained at different times are shown in Figure 10. In this case, it was possible to notice that crystals showed a growing trend for over 5 days without signs of degradation. This makes clear that, in order to use a Nafion^®^ 117 membrane as nucleation support for HEWL crystallization, the previous proton exchange process becomes essential to avoid pH-driven degradation of the protein crystals.

**Figure 11:**
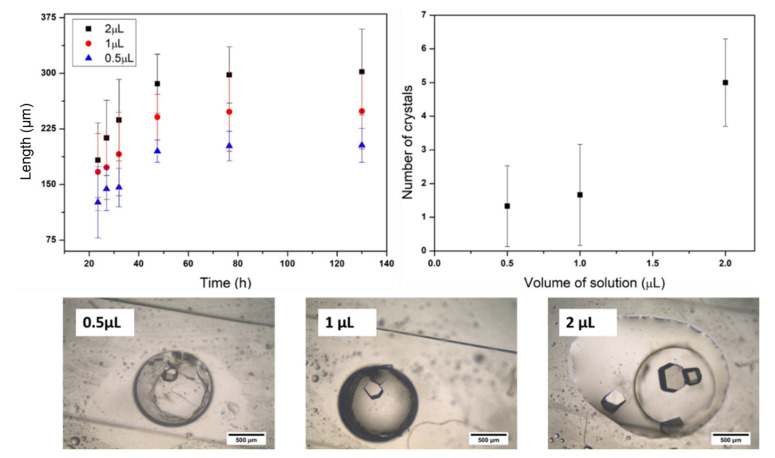
Crystal growth kinetics (**top left**) and the number of crystals per volume of solution (**top right**). On the bottom: crystals grown in different volumes of solution, observed after 130 h.

Reproducibility of the crystallization results was assessed for different volumes of protein solution in the well-type microdevice. Each condition was repeated nine times. Figure 11 displays the results related to the length and the number of crystals obtained, using different volumes of the crystallization solution, varying from 0.5 to 2.0 μL but maintaining the same membrane contact area (due to Nafion membrane hydrophobicity, small volumes of protein solution may be accommodated on the membrane on a surface area smaller than the well diameter, speeding-up the diffusion rate of the process; however, the volumes used in this experiment allowed for all the drops to completely wet the well surface area. Even though the final equilibrium condition and the water transport rate through the membrane were supposedly the same, the number and size of crystals increased with the volume of the solution dispensed in the well-type microdevice. The higher number of crystals may be attributed to the higher amount of protein available for nucleation and crystal growth. In fact, no differences were found in the time required for the first crystals to appear. This behavior is probably due to the low time shift for reaching nucleation conditions among the different volumes tested. 

In general, it is possible to conclude that the designed micro-device allows for the control of the crystals’ number and size, by changing the volume of the protein solution dispensed in the well-type micro-contactor. The crystals obtained showed high stability for over 5 days.

### 3.4. X-ray Diffraction and Structure Determination

Data collection, processing, and refinement are reported in Table 2. The crystals diffracted to a maximum resolution of 1.7 Å. The collected, indexed, and integrated data were scaled and merged using the software pipeline in PROTEUM3 (Bruker AXS 2015). 

**Figure 12:**
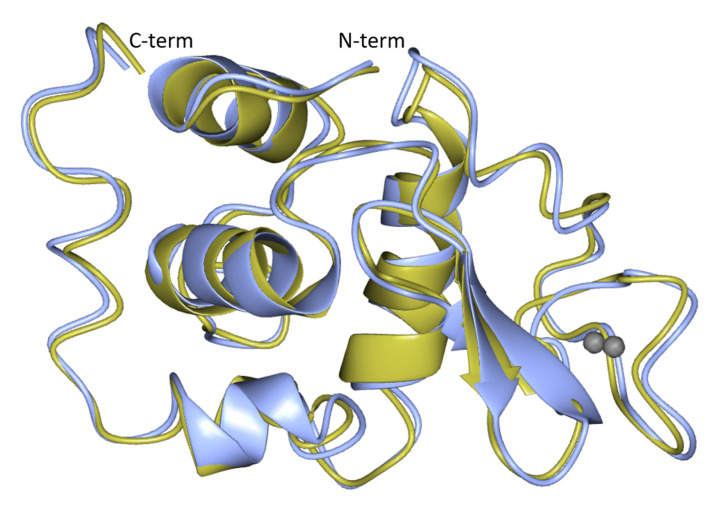
Ribbon representation of HEWL protein. The model obtained by molecular replacement using the in-house collected data (blue) is superposed on the known structure of HEWL (yellow) (pdb code: 3a8z). The grey spheres correspond to Na^+^ atoms. The superposition of the pdb model and the calculated structure generate an rmsd (root-mean-square deviation of atomic position) of 0.83 Å for 129 α carbon atoms. The picture was produced by using the program CCP4mg.

The analyzed crystals belong to space group *P*4_3_2_1_2 (the space group gives an indication of the type of symmetry of the crystal; this space group corresponds to a tetragonal system where the unit cell dimensions a, b, and c are a = b ≠ c [44]. HEWL crystals conventionally belong to this space group). The diffraction data of the crystals are characterized by a low R-merge value, high signal-to-noise ratio, and a completeness of 98.7%. The electron density map was generated after the structure solution by molecular replacement (MR) using 3a8z as a reference structure. The R-work/R-free ratio (R-work is a parameter that measures the deviation in the predicted model from reality, and for a well-resolved structure, it should be <0.200; R-free is a parameter used to assess the overfitting of experimental data and should not excessively differ from R-work [45]) after refinement was lowered to 0.177/0.216. According to Ramachandran statistics analysis (used to assess stereochemical quality of the protein model), 93.97% of the residues were found in favored regions (energetically favored values of the molecular torsion angles), 6.03% were found in allowed regions, and no outlier residues were found. A ribbon representation of the HEWL molecule is displayed in Figure 12. Summarizing, all the parameters evaluated in Table 2 and described in this section are indicators of high diffraction quality.

Additionally, in the case of completely unknown structures, where it is not possible to select a searching model for applying the MR method for resolving the phase problem, derivatization of the crystals might be performed using the same microdevice, allowing, in this way, the possibility to approach the phase problem via performing isomorphous replacement methods. In these cases, the derivatization process can be controlled by the selective diffusion of ions across the membrane, avoiding abrupt changes in the local environment and handling of the crystals [20]. Indeed, simulation of the transport of Hg^2+^ in the microdevice showed how it is possible to control the rate of ion diffusion by changing the volume of protein solution.

## 4. Conclusions

Trial and error is still the leading strategy for finding conditions for protein crystallization and for crystals derivatization, and hundreds (or thousands) of experimental conditions usually need to be tried until suitable crystals are obtained; very often, the amount of available pure protein is a limiting factor. Microfluidics technology provides advantages to the protein crystallization field with several designs that allow for a lower consumption of reagents and a higher number of trials. In addition, membrane technology concurs with the control of supersaturation, as well as the diffusion rates of derivatizing agents needed to obtain crystals with a high diffraction quality. In this work, a Nafion^®^ membrane was integrated with a polydimethylsiloxane (PDMS) microdevice for protein crystallization. Functionality of the device was tested and proved to allow for a successful crystallization of Hen Egg White Lysozyme (HEWL) and subsequent in situ and gentle crystal derivatization [20] based on an efficient control of the diffusion rates of the derivatizing agent (Hg^2+^ was used as a model agent in the present study), assured by an ion exchange membrane (Nafion^®^ 117) integrated in the microdevice. These conditions were key to assure crystal structural integrity along the derivatization process, proving to be a more efficient alternative to the traditional crystal soaking methodologies. This work provides insights into the impact of the membrane (Nafion^®^ membrane)-regulated mass transport of water and derivatizing agents on protein crystallization and crystal derivatization, highlighting the importance of mass transport studies for an enhanced design of microfluidic devices for crystallization. The results obtained unveiled the dependence of the size and number of crystals on the volume of protein solution, showing that it is possible to predefine the desirable crystal structural parameters by suitable selection of the solution volume in the microdevice wells. The crystals grown in the micro-device were harvested and analyzed by X-rays showing a high diffraction quality.

Furthermore, this work shows the importance of implementing microfabrication methodologies that assure the chemical stability and inertness of the materials used for the fabrication of devices for crystallization and crystal derivatization. The release of compounds from the crystallization device may change the physicochemical conditions of the processing media, such as changes in pH to extreme values (as observed in the present work), affecting the structural integrity of the crystals.

Finally, showing the feasibility of the microfluidic device for subsequent successful crystallization and derivatization processes, this work paves the way for exploring additional advantages offered by this microfluidic system. In particular, the presence of the high number of wells (75 wells) and individualized channels is expected to allow for a simultaneous screening of a high number of different conditions (75 different conditions), allowing for faster, cheaper, and high-throughput crystal formation with the diffracting quality required for a more efficient crystal structure resolution.

## Figures and Tables

**Table 1:** Mass transfer coefficient for water and Hg^2+^.

Substance	Mass Transfer Coefficient (m/s)
Water	$4.9\times 10^{-6}$
Hg^2+^	$1.9\times 10^{-9}$

**Table 2:** Statistics of X-ray diffraction data collection and automated model building and refinement for HEWL crystals (values for the last resolution shell are in parentheses).

**X-ray Diffraction Parameters and Statistics**	
space group	*P* 4_3_ 2_1_ 2
wavelength (Å)	1.5418
resolution range (Å)	22.20–1.70 (1.80–1.70)
unit cell parameters (Å) *a*, *b*, *c*	78.6, 78.6, 36.9
total reflections	147469 (9518)
unique reflections	13166 (1981)
multiplicity	11.2 (4.8)
completeness (%)	98.7 (94.3)
mean I/sigma (I)	18.8 (2.1)
R-merge ^†^	0.100 (0.662)
R-sigma ^+^	0.055 (0.485)
**Model Building and Refinement**	
R-work ^‡^/R-free *	0.177/0.216
N of nonhydrogen atoms	1226
N of macromolecule atoms	1058
N of protein residues	129
N of Sodium atoms	1
N of Chloride atoms	7
N of water molecules	136
RMSD (bonds) (Å)	0.009
RMSD (angles) (°)	1.589
Ramachandran favored (%)	93.97
Ramachandran allowed (%)	6.03
Ramachandran outliers (%)	0.00
Average B-factor (Å^2^) main chain	13.5
Average B-factor (Å^2^) side chain	16.2
Average B-factor (Å^2^) for Na^+^	23.3
Average B-factor (Å^2^) for Cl^−^	27.5
Average B-factor (Å^2^) for waters	24.9

^†^ $R_{\mathrm{merge}}=\frac{\sum_{hkl}\sum_{i=1}^{n}\left|I_{i}\left(hkl\right)-\bar{I}\left(hkl\right)\right|}{\sum_{hkl}\sum_{i=1}^{n}I_{i}\left(hkl\right)}$, where I is the observed intensity and $\bar{I}$ is the statistically weighted average intensity of multiple observations; ^+^$\;R_{\mathrm{sigma}}=\frac{\sum_{hkl}\sqrt{1/\left(n-1\right)}\sum_{i=1}^{n}\left|I_{i}\left(hkl\right)-\bar{I}\left(hkl\right)\right|}{\sum_{hkl}\sum_{i=1}^{n}I_{i}\left(hkl\right)}$, a redundancy-independent version of $R_{\mathrm{merge}}$; ^‡^$\;R_{\mathrm{work}}=\frac{\sum_{hkl}\left|\left|F_{obs}\left(hkl\right)\right|-\left|F_{calc}\left(hkl\right)\right|\right|}{\sum_{hkl}\left|F_{obs}\left(hkl\right)\right|}$, where $\left|F_{calc}\right|$ and $\left|F_{obs}\right|$ are the calculated and observed structure factor amplitudes, respectively; * *R-free* is calculated for a randomly chosen 5% of the reflections.

## Data Availability

Data available on request.

## Supplementary Materials

The following are available online at https://www.mdpi.com/article/10.3390/membranes11080549/s1, Figure S1: Conductivity and NaCl concentration over time in the diffusion cell; Figure S2: Driving force versus time; Figure S3: Conductivity and NaCl concentration over time in compartment A; Figure S4: Amount of NaCl crossing the membrane over time.

## References

[1] Gavira J.A. (2016). Current trends in protein crystallization. Arch. Biochem. Biophys..

[2] Li L., Ismagilov R.F. (2010). Protein crystallization using microfluidic technologies based on valves, droplets, and SlipChip. Annu. Rev. Biophys..

[3] Abdallah B.G., Roy-Chowdhury S., Fromme R., Fromme P., Ros A. (2016). Protein Crystallization in an Actuated Microfluidic Nanowell Device. Cryst. Growth Des..

[4] Du W., Li L., Nichols K.P., Ismagilov R.F. (2009). SlipChip. Lab Chip..

[5] Wang L., Sun K., Hu X., Li G., Jin Q., Zhao J. (2015). A centrifugal microfluidic device for screening protein crystallization conditions by vapor diffusion. Sens. Actuators B Chem..

[6] Yu Y., Wang X., Oberthür D., Meyer A., Perbandt M., Duan L., Kang Q. (2012). Design and application of a microfluidic device for protein crystallization using an evaporation-based crystallization technique. J. Appl. Crystallogr..

[7] De Wijn R., Hennig O., Roche J., Engilberge S., Rollet K., Fernandez-Millan P., Brillet K., Betat H., Mörl M., Roussel A. (2019). A simple and versatile microfluidic device for efficient biomacromolecule crystallization and structural analysis by serial crystallography. IUCrJ.

[8] Curcio E., Criscuoli A., Drioli E. (2001). Membrane crystallizers. Ind. Eng. Chem. Res..

[9] Di Profio G., Curcio E., Cassetta A., Lamba D., Drioli E. (2003). Membrane crystallization of lysozyme: Kinetic aspects. J. Cryst. Growth..

[10] Di Profio G., Curcio E., Drioli E. (2005). Trypsin crystallization by membrane-based techniques. J. Struct. Biol..

[11] Simone S., Curcio E., Di Profio G., Ferraroni M., Drioli E. (2006). Polymeric hydrophobic membranes as a tool to control polymorphism and protein ligand interactions. J. Membr. Sci..

[12] Di Profio G., Polino M., Nicoletta F.P., Belviso B.D., Caliandro R., Fontananova E., De Filpo G., Curcio E., Drioli E. (2014). Tailored hydrogel membranes for efficient protein crystallization. Adv. Funct. Mater..

[13] Pike A.C.W., Garman E.F., Krojer T., Delft F., von Carpenter E.P. (2016). An overview of heavy-atom derivatization of protein crystals. Acta Crystallogr. Sect. D Struct. Biol..

[14] Taylor G.L. (2010). Introduction to phasing. Acta Crystallogr. Sect. D Biol. Crystallogr..

[15] Dauter M., Dauter Z. (2007). Phase determination using halide ions. Methods Mol. Biol..

[16] Morth J.P., Sørensen T.L.M., Nissen P. (2006). Membrane’s eleven: Heavy-atom derivatives of membrane-protein crystals. Acta Crystallogr. Sect. D Biol. Crystallogr..

[17] Giacovazzo C., Ladisa M., Siliqi D. (2002). The approach of the joint probability distribution functions: The SIR-MIR, SAD-MAD and SIRAS-MIRAS, cases, Zeitschrift Für Krist. Cryst. Mater..

[18] Joyce M.G., Radaev S., Sun P.D. (2010). A rational approach to heavy-atom derivative screening. Acta Crystallogr. Sect. D Biol. Crystallogr..

[19] Agniswamy J., Joyce M.G., Hammer C.H., Sun P.D. (2008). Towards a rational approach for heavy-atom derivative screening in protein crystallography. Acta Crystallogr. Sect. D Biol. Crystallogr..

[20] Polino M., Carvalho A.L., Juknaite L., Portugal C.A.M., Coelhoso I.M., Romão M.J., Crespo J.G. (2017). Ion-Exchange Membranes for Stable Derivatization of Protein Crystals. Cryst. Growth Des..

[21] Xu T. (2005). Ion exchange membranes: State of their development and perspective. J. Memb. Sci..

[22] Galinha C.F., Carvalho G., Portugal C.A.M., Guglielmi G., Reis M.A.M., Crespo J.G. (2012). Multivariate statistically-based modelling of a membrane bioreactor for wastewater treatment using 2D fluorescence monitoring data. Water Res..

[23] Qin D., Xia Y., Whitesides G.M. (2010). Soft lithography for micro- and nanoscale patterning. Nat. Protoc..

[24] Li X., Feng F., Zhang K., Ye S., Kwok D.Y., Birss V. (2012). Wettability of Nafion and Nafion/Vulcan Carbon Composite Films. Langmuir.

[25] Xia Y., Whitesides G.M. (1998). Soft-lithography. Angew. Chem. Int. Ed..

[26] Pimpin A., Srituravanich W. (2012). Review on Micro- and Nanolithography Techniques and their Applications. Eng. J..

[27] Phan D.-T., Yang C., Nguyen N.-T. (2017). A sugar-template manufacturing method for microsystem ion-exchange membranes. J. Micromech. Microeng..

[28] Yuen P.K., Su H., Goral V.N., Fink K.A. (2011). Three-dimensional interconnected microporous poly(dimethylsiloxane) microfluidic devices. Lab Chip..

[29] Phan D.-T., Yang C., Nguyen N.-T. (2015). Fabrication of nanoporous junctions using off-the- shelf Nafion membrane. J. Micromech. Microeng..

[30] Slouka Z., Senapati S., Chang H.-C. (2014). Microfluidic Systems with Ion-Selective Membranes. Annu. Rev Anal. Chem..

[31] Pham M.H., Barz D.P.J. (2017). Bonding Nafion® with polydimethysiloxane: A versatile approach towards ion-exchange membrane microfluidic devices. J. Membr. Sci..

[32] Jung H., Won J. (2012). Role of the glass transition temperature of Nafion 117 membrane in the preparation of the membrane electrode assembly in a direct methanol fuel cell (DMFC). Int. J. Hydrogen Energy.

[33] Pessoa-Lopes M., Crespo J.G., Velizarov S. (2016). Arsenate removal from sulphate-containing water streams by an ion-exchange membrane process. Sep. Purif. Technol..

[34] Potterton L., Agirre J., Ballard C., Cowtan K., Dodson E., Evans P.R., Jenkins H.T., Keegan R., Krissinel E., Stevenson K. (2018). CCP 4 i 2: The new graphical user interface to the CCP 4 program suite research papers. Acta Crystallogr. Sect. D Struct. Biol..

[35] Hauptman H. (1997). Phasing methods for protein crystallography. Curr. Opin. Struct. Biol..

[36] Evans P., McCoy A. (2007). An introduction to molecular replacement. Acta Crystallogr. Sect. D Biol. Crystallogr..

[37] RCSB PBD: Protein Data Bank. https://www.rcsb.org/structure/3A8Z.

[38] Emsley P., Cowtan K. (2004). Coot: Model-building tools for molecular graphics research papers. Acta Crystallogr. Sect. D Biol. Crystallogr..

[39] Murshudov G.N., Skubák P., Lebedev A.A., Pannu N.S., Steiner R.A., Nicholls R.A., Winn M.D., Long F., Vagin A.A. (2011). REFMAC5 for the refinement of macromolecular crystal structures. Acta Crystallogr. Sect. D Biol. Crystallogr..

[40] Cowtan K. (2006). The Buccaneer software for automated model building. 1. Tracing protein chains. Acta Crystallogr. Sect. D Biol. Crystallogr..

[41] McNichola S., Potterton E., Wilson K.S., Noble M.E.M. (2011). Presenting your structures: The CCP4mg molecular-graphics software. Acta Crystallogr. Sect. D Biol. Crystallogr..

[42] Chen V.B., Arendall W.B., Headd J.J., Keedy D.A., Immormino R.M., Kapral G.J., Murray L.W., Richardson J.S., Richardson D.C. (2010). MolProbity: All-atom structure validation for macromolecular crystallography. Acta Crystallogr. Sect. D Biol. Crystallogr..

[43] Iwai W., Yagi D., Ishikawa T., Ohnishi Y., Tanaka I., Niimura N. (2008). Crystallization and evaluation of hen egg-white lysozyme crystals for protein pH titration in the crystalline state. J. Synchrotron Rad..

[44] Wukovitz S.W., Yeates T.O. (1995). Why protein crystals favour some space-groups over others. Nat. Struct. Biol..

[45] Bruger A.T. (1992). Free R value: A novel statistical quantity for assessing the accuracy of crystal structures. Nature.

