# Supplementary material for: Protein Crystallization in a Microfluidic Contactor with Nafion®117 Membranes
Source: Membranes (Basel). 2021 Jul 21;11(8):549. doi: 10.3390/membranes11080549 (PMC8398885; doi:10.3390/membranes11080549)
Supplement: Supplementary file 1 [file membranes-11-00549-s001.zip › membranes-1279682-supplementary.pdf]

Supporting Information

Protein Crystallization in a Microfluidic Contactor with Nafion®117 Membranes

M. Polino[1], H. S. Rho[2], M. P. Pina[3,4,5], R. Mallada[3,4,5], A. L. Carvalho[6,7], M. J. Romão[6,7], Isabel Coelhoso[1], J. G. E. Gardeniers[8,*], J. G. Crespo[1], Carla A. M. Portugal[1,*]

[1] LAQV-REQUIMTE, Department of Chemistry, NOVA School of Science and Technology, Universidade Nova de Lisboa, 2829-516 Caparica, Portugal

[2] Department of Instructive Biomaterials Engineering, MERLN Institute for Technology-Inspired Regenerative Medicine, Maastricht University, 6229 ER Maastricht, The Netherlands

[3] Instituto de Nanociencia y Materiales de Aragón (INMA), Universidad de Zaragoza-CSIC, 50009 Zaragoza, Spain

[4] Department of Chemical & Environmental Engineering, University of Zaragoza, 50018 Zaragoza, Spain

[5] Networking Research Centre on Bioengineering, Biomaterials and Nanomedicine, CIBER-BBN, 28029 Madrid, Spain

[6] UCIBIO – Applied Molecular Biosciences Unit, Department of Chemistry, School of Science and Technology, NOVA University Lisbon, 2819-516 Caparica, Portugal

[7] Associate Laboratory i4HB - Institute for Health and Bioeconomy, School of Science and Technology, NOVA University Lisbon, 2819-516 Caparica, Portugal

[8] Mesoscale Chemical Systems, TNW Faculty, University of Twente, P.O. Box 217, 7500 AE Enschede, The Netherlands

***Corresponding authors:**

Carla A. M. Portugal

LAQV-REQUIMTE, Department of Chemistry

FCT-Universidade Nova de Lisboa

Campus da Caparica

2829-516 Caparica

Email: cmp@fct.unl.pt

J. G. E. Gardeniers

Mesoscale Chemical Systems

TNW-MCS, Carré CR1349

MESA+ Institute - University of Twente

P.O. Box 217, 7500 AE Enschede, The Netherlands

Email: j.g.e.gardeniers@utwente.nl

NaCl transport across Nafion®

When a cation-exchange membrane (as Nafion®) contacts a pure water solution on one side and a salt solution on the other side, water will move from the water compartment to the salt solution compartment until the osmotic pressure is equilibrated. However, due to the high osmotic pressure difference and the absence of a cation in the water compartment to be exchanged with Na^+, a leak of NaCl is expected (electrolyte leakage)[1].

A

B)

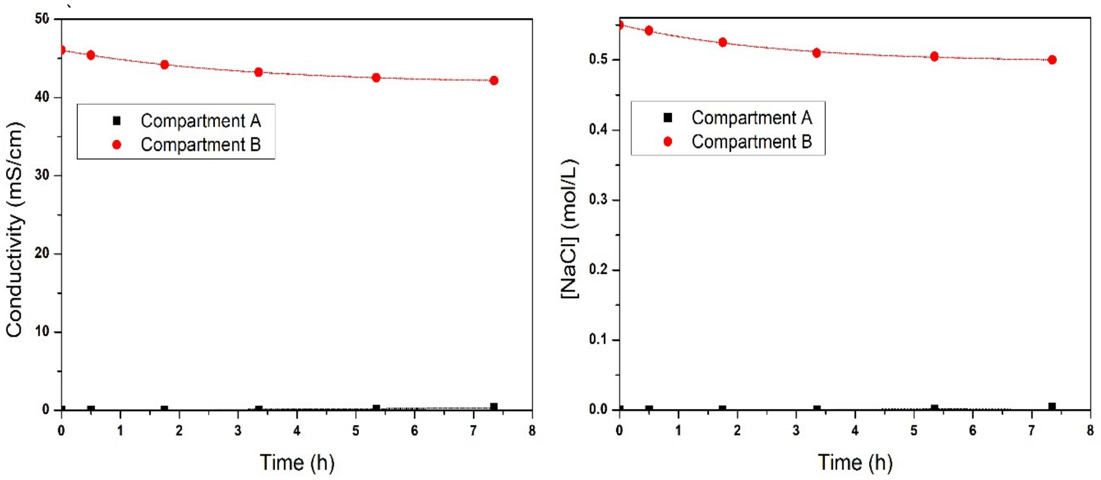

Figure S1 (**A**) Conductivity over time in the diffusion cell; (**B**): NaCl concentration over time in compartment A and B of the diffusion cell

In order to assess the entity of the leak, conductivity of the solutions in the compartment A and B of the diffusion cell in Figure 3a of the main text was followed over time (Figure S1.A). By means of equipment calibration, the conductivity values were converted into NaCl concentration (Figure S1.B).

Mass transfer coefficient of NaCl

From the difference between NaCl concentration in compartment A and B the driving force over time was calculated and represented in Figure S2.

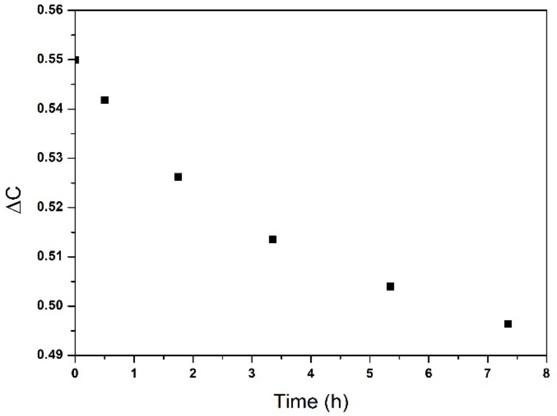

Figure S2 Driving force versus time

The variation of driving force over the time interval of measurement has been calculated to be 0.02M (4% of the average driving force). For this reason, it was considered constant for the calculations of water mass transfer coefficient.

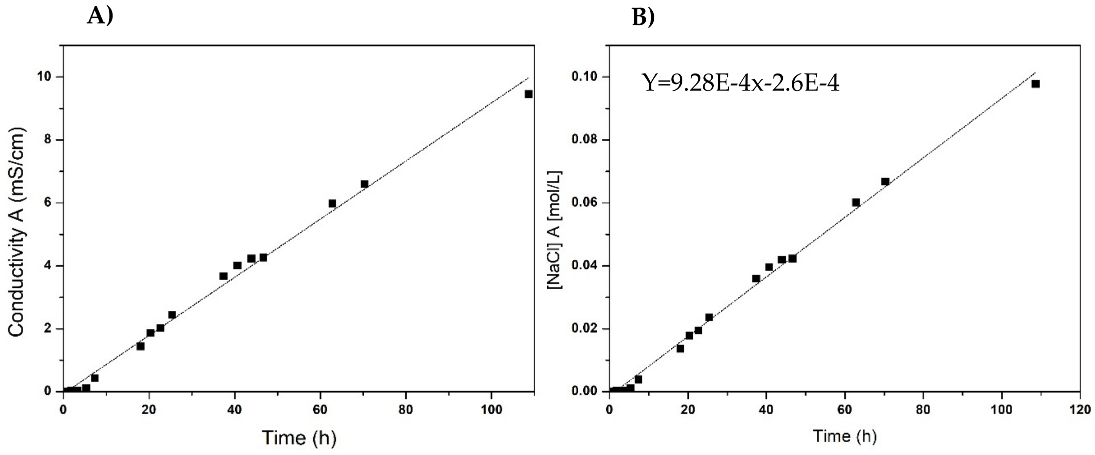

Figure S3 (**A**) Conductivity over time in compartment A; (**B**) NaCl concentration over time in compartment A

In Figure S3 the change of conductivity (A) and NaCl concentration (B) in compartment A over time is displayed. The change of concentration is due to both, increased concentration of NaCl in compartment A and decreased water volume due to its transport to compartment B. Therefore, the flux ($9.28x10^{-4}$mol/L.h) calculated by fitting this curve can be considered apparent.

The real amount of NaCl crossing the membrane has been calculated by multiplying the [NaCl] over time by the corresponding volume of the compartment over time and shown in Figure S4.

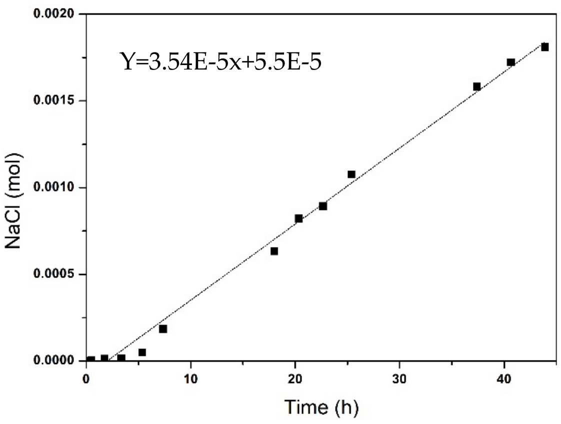

Figure S4 Amount of NaCl crossing the membrane over time

From Figure S4, by dividing the slope of the curve by the area of the membrane (A), the molar flux of NaCl (J_{NaCl}) was calculated (Equation. 1).

$$J_{NaCl} = \frac{mol_{NaCl}}{tA} \quad (1)$$

The J_{NaCl} can be also defined as:

$$J_{NaCl} = K_{NaCl}\Delta C \quad (2)$$

Where K_{NaCl} is the apparent mass transfer coefficient for NaCl and ΔC is the NaCl concentration difference between the two sides of the membrane. Also in this case, due to the minimal variation of driving force ΔC was considered constant over time (0.52M)

Hence, K_{NaCl} was calculated as:

$$K_{NaCl} = \frac{J_{NaCl}}{\Delta C} \quad (3)$$

The value for K_{NaCl} was: 3.92*10^{-8}m/s. This value is two orders of magnitude lower compared to the water flux suggesting that the variation of concentration is mostly determined by the water transport.

References

[1] M. Pessoa-Lopes, J.G. Crespo, S. Velizarov, Arsenate removal from sulphate-containing water streams by an ion-exchange membrane process, Sep. Purif. Technol. 166 (2016) 125–134.
